# Supplementary material for: Suggested role of NosZ in preventing N2O inhibition of dissimilatory nitrite reduction to ammonium
Source: mBio. 2023 Sep 22;14(5):e01540-23. doi: 10.1128/mbio.01540-23 (PMC10653820; doi:10.1128/mbio.01540-23)
Supplement: Supplemental materials — Texts S1 and S2, Fig. S1 to S4, and Tables S1 to S3. [file mbio.01540-23-s0001.pdf]

Supplemental materials

Suggested role of NosZ in preventing N_2O inhibition of dissimilatory nitrite reduction to ammonium

Sojung Yoon[a], Hokwan Heo[a], Heejoo Han[a], Dong-Uk Song[a], Lars R. Bakken[b], Åsa Frostegård[b], Sukhwan Yoon [a#]

[a] Department of Civil and Environmental Engineering, Korea Advanced Institute of Science and Technology (KAIST), Daejeon, South Korea

[b] Faculty of Chemistry, Biotechnology and Food Science, Norwegian University of Life Science, Ås, Norway

Running title: N_2O inhibition of DNRA and its relief by NosZ

[#]Address correspondence to Sukhwan Yoon, syoon80@kaist.ac.kr

Supplemental methods and materials

Text S1. Stoichiometric calculation of additional electron-accepting capacity gained by NosZ activity.

Assuming that 95% of NO_2^- is reduced to NH_4^+ via DNRA (Eq. 1) and 5% results in N_2O production (Eq. 2), the number of moles of electrons transferred to N_2O for reduction to N_2 would be 0.05 per mole NO_2^- consumed, amounting to 0.88% of the number of moles of electrons transferred to NH_4^+ from 0.95 mole NO_2^- (5.7 per mole NO_2^- consumed).

$$NO_2^- + 6e^- + 8H^+ \rightarrow NH_4^+ + 2H_2O \quad (1)$$

$$N_2O + 2e^- + 2H^+ \rightarrow N_2 + H_2O \quad (2)$$

Text S2. Reverse transcription quantitative PCR (RT-qPCR) method

The *nrfA* and *nosZ* transcripts in *Bacillus* sp. DNRA2 cultures were quantified by RT-qPCR using a previously established protocol (1). Immediately after sampling, 1 mL of RNA Protect Bacteria Reagent (Qiagen, Hilden, Germany) was mixed with the 0.5-mL culture in a RNase-free 1.5-mL Safe-Lock tube (Eppendorf, Hamburg, Germany). The cell pellets collected after 5 min centrifugation at 5,000 x g were stored at -80 °C until use. The pellets were thawed on ice, and 350 µL buffer RLT was added to the thawed tube along with 40 mg of 0.1 mm diameter glass beads (Omni International, Kennesaw, GA). The cells were disrupted using an Omni Bead Ruptor 12 homogenizer (Kennesaw, GA), and total RNA was extracted using RNeasy Mini Kit (Qiagen) following the protocol provided by the manufacturer. After digestion with RNase-Free DNase Set (Qiagen), RNA was purified with RNeasy MinElute Cleanup kit (Qiagen). The absence of genomic DNA in the eluent was later confirmed by performing PCR targeting the *recA* gene with a fraction of the eluent stored at -80 °C. Reverse transcription of total RNA was performed using Superscript® III Reverse Transcriptase (Invitrogen, Carlsbad, CA). The remaining RNA was removed using RNase H (Invitrogen), and the resulting cDNA solution was diluted 5-fold with nuclease-free water (Invitrogen) and stored at -20 °C. The qPCR assays were performed using SYBR Green detection chemistry, using the primer sets designed de novo from the *nrfA* and *nosZ* gene sequences downloaded from the NCBI's genome database (Accession number: NZ_JABAIT000000000) with Primer3 software (Table S3; Rozen and Skaletsky, 2000). The single-copy housekeeping gene recA was also quantified, as this gene is known to be constitutively expressed at a relatively constant level, and thus has been used for normalization of RT-qPCR data in previous studies (2, 3). The information regarding the primer sets is summarized in Table S3. Quantitative PCR was performed on a QuanStudio 3 real-time PCR instrument (Thermo Fisher Scientific, Waltham, MA). For each target gene, a calibration curve was constructed with a 108-101 copies µL-1 dilution series of the amplicons inserted in PCR®2.1 vectors.

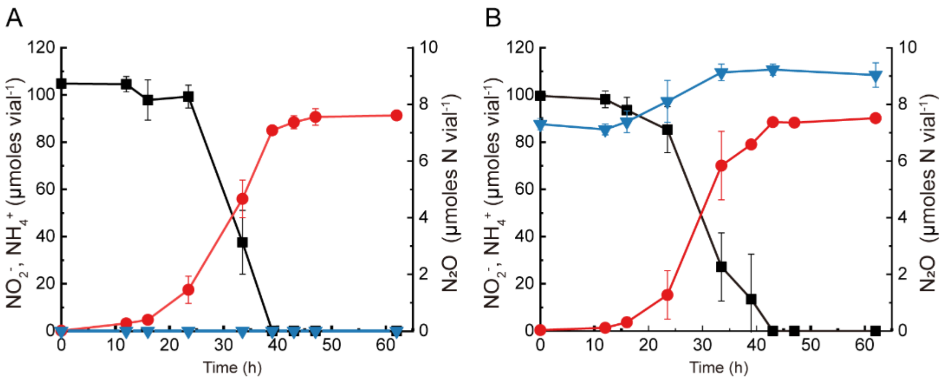

FIG S1. Anaerobic incubation of *Bacillus* sp. DNRA2 cultures with 1.0 mM NO_2^-. (A) Control cultures without N_2O or C_2H_2 in the headspace, and (B) cultures amended with N_2O (3.5 µmoles per vial) and C_2H_2 (10% v/v in the headspace), corresponding to Fig. 1A and 1D, respectively. The data points represent the average of values obtained from triplicate cultures and the error bars their standard deviations (■, NO_2^- ; ●, NH_4^+ ; ▼, N_2O-N).

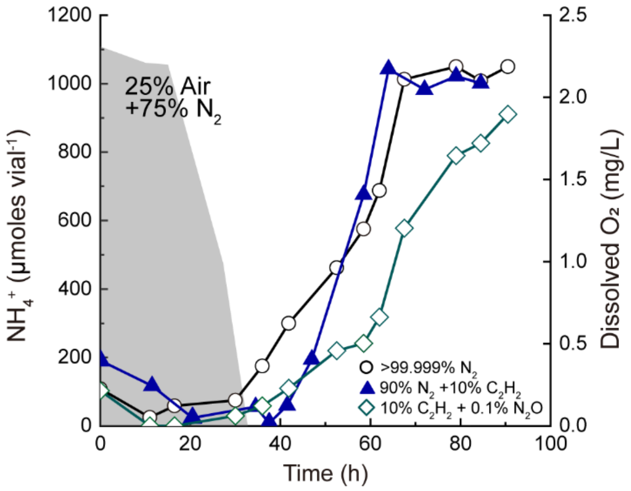

FIG S2. Independent replicate of the batch reactor experiment, the result of which was presented in Fig. 3.

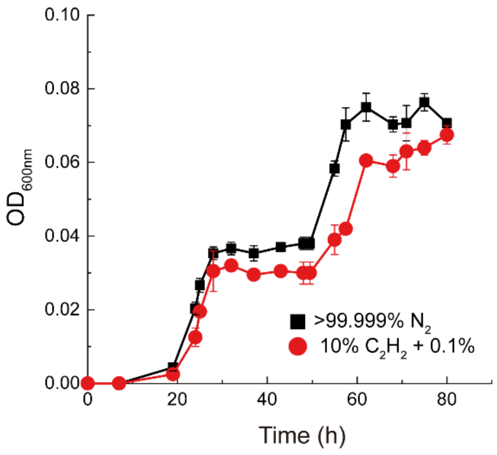

FIG S3. Microbial growth monitored during the batch incubation experiments performed with headspace replenishments (presented in Fig. 5). The red (●) and black (■) symbols represent the data collected from cultures incubated with N_2O and C_2H_2 amendment and without, respectively. The data points represent the average of values obtained from triplicate cultures and the error bars their standard deviations.

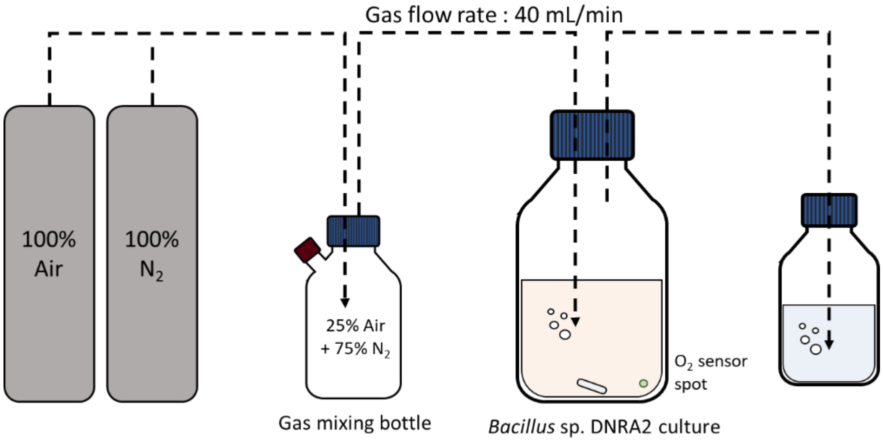

FIG S4. Schematic depiction of the batch reactor used for isolating the effect of C_2H_2 from that of N_2O

Table S1. Inventories of *nrfA*/*nosZ* genes in sequenced *Bacillus* genomes

***Bacillus* spp.**	**Accession number**	**NrfA**	**NrfA Similarity / Identity (%)**	**NosZ**	**NosZ Similarity / Identity (%)**
Bacillus sp. FJAT-27445	LMTI00000000	WP_059171458.1	83/74	WP_059172528.1	93/86
Bacillus sp. FJAT-18017	CP012602	WP_053598655.1	85/77	WP_053601334.1	93/85
Bacillus sp. FJAT-27245	LIGI00000000	WP_053367445.1	82/73	N/A[a]	N/A[a]
Bacillus sp. B-jedd	CCXR00000000	WP_048826161.1	82/73	N/A[a]	N/A[a]
Bacillus sp. EB01	CCBA000000000	WP_043934052.1	85/77	N/A[a]	N/A[a]
Bacillus sp. UNC41MFS5	JMLP00000000	WP_026563249.1	82/73	WP_155928933.1	90/82
Bacillus sp. 1NLA3E	CP005586	WP_015595323.1	88/78	N/A[a]	N/A[a]
Bacillus sp. Marseille-P3661	OESV00000000	WP_102345480.1	81/70	WP_102345060.1	88/78
Bacillus massiliigabonensis Marseille-P2639	FZRJ00000000	WP_102274198.1	88/76	WP_102273543.1	86/73
Bacillus sp. V3-13	PGUZ00000000	WP_101657743.1	88/79	WP_101658101.1	92/86
Bacillus sp. T33-2	PGVB00000000	WP_101581007.1	89/82	N/A[a]	N/A[a]
Bacillus sp. AFS031507	NUOW00000000	WP_098933286.1	82/73	WP_098932351.1	90/82
Bacillus sp. AFS073361	NUZU00000000	WP_098574575.1	82/72	WP_098571583.1	90/83
Bacillus sp. 7884-1	NPDD00000000	WP_095250826.1	83/74	WP_095247464.1	93/85
Bacillus sp. OK048	FNHN00000000	WP_090759355.1	82/73	WP_090762522.1	93/84
Bacillus sp. OV166	FXWM00000000	WP_088089315.1	82/73	WP_088084798.1	90/82
Bacillus shivajii strain JCM 32183	CP084703	WP_226518355.1	70/55	WP_226517035.1	81/70
Bacillus sp. sid0103	JAHUWN000000000	WP_218349315.1	82/72	WP_218346968.1	90/83
Bacillus sp. FJAT-29790	JAHNZZ000000000	WP_217040143.1	88/79	N/A[a]	N/A[a]
Bacillus sp. ISL-75	JAGGQX000000000	WP_215010644.1	81/73	N/A[a]	N/A[a]
Bacillus sp. ISL-41	JAGGQP000000000	WP_214739878.1	87/78	WP_214741780.1	85/74
Bacillus sp. ISL-35	JAGGQK000000000	WP_214709187.1	86/78	WP_214705222.1	85/74
Bacillus sp. SM2101	JAEUFG000000000	WP_214480373.1	77/65	N/A[a]	N/A[a]

Bacillus sp. FJAT-50051	JAGYPE000000000	WP_213144551.1	82/72	WP_213147918.1	91/83
Bacillus sp. REN3	JAERSD000000000	WP_210364851.1	88/77	WP_210364329.1	86/74
Bacillus sp. B15-48	WOFU00000000	WP_203554797.1	86/76	WP_203553798.1	91/83
Bacillus renqingensis strain REN2	JADDIU000000000	WP_199420352.1	82/74	WP_199418569.1	92/83
Bacillus sp. EB106-08-02-XG196	JABWSY000000000	WP_179160110.1	82/74	WP_179157987.1	93/84
Bacillus sp. DNRA2	JABAIT000000000	WP_169101734.1	100/100	WP_169101635.1	100/100
Bacillus vireti LMG 21834	ALAN00000000	WP_024030559.1	83/74	ETI67672.1	83/77
Bacillus sp. MM2020_1	JAANMP000000000	WP_166258001.1	82/73	WP_166257289.1	86/77
Bacillus sp. S3	CP039727	WP_149871314.1	82/73	WP_149872441.1	91/82
Bacillus marasmi strain Marseille-P3556	CABHPS000000000	WP_147532674.1	88/77	WP_147535054.1	97/94
Bacillus sp. X1(2014)	VEED00000000	WP_144555208.1	83/74	WP_144551817.1	91/83
Bacillus dakarensis strain Marseille-P3515	FTOZ00000000	WP_139339527.1	86/74	WP_077215134.1	93/85
Bacillus marinisedimentorum strain NC2-31	LWBL00000000	WP_070120484.1	86/73	WP_070121385.1	90/81
Bacillus sp. FJAT-29814	LMTJ00000000	WP_066310973.1	81/71	WP_066310293.1	91/82
Bacillus sp. FJAT-27225	MAYU00000000	WP_066197006.1	85/77	N/A[a]	N/A[a]
Bacillus dafuensis strain FJAT-25496	CP042593	WP_057772228.1	86/75	N/A[a]	N/A[a]
Bacillus sp. A116_S68	CP058292	UJW55953.1	69/57	N/A[a]	N/A[a]
Bacillus selenitireducens MLS10	CP001791	ADH98815.1	68/51	N/A[a]	N/A[a]
Bacillus sp. AFS006103	NTXX00000000	PEQ95703.1	81/73	PEQ96906.1	86/76
Bacillus sp. MRMR6	MSLS00000000	WP_075689611.1	82/72	N/A[a]	N/A[a]
Bacillus sp.17376	AWXY00000000	ESU30961.1	87/78	N/A[a]	N/A[a]
Bacillus sp. FJAT-21945	LITN00000000	KOP83814.1	87/76	KOP71743.1	86/73

[a] N/A, not applicable

Table S2. Lactate concentrations measured before and after incubation

		w/o amendment (A)	N_2O-amended (B)	C_2H_2-amended (C)	C_2H_2- and N_2O- amended (D)
***Bacillus* sp. DNRA2 incubation with 1 mM NO_2^- (Fig. 1)**	**Initial (mM)**	5.03 (0.03)[a]	5.02 (0.003)[a]	5.04 (0.02)[a]	5.04 (0.03)[a]
	**Final (mM)**	3.32 (0.11)[a]	3.23 (0.05)[a]	3.57 (0.10)[a]	3.68 (0.20)[a]
***Bacillus* sp. DNRA2 incubation with 1 mM NO_3^- (Fig. 2)**	**Initial (mM)**	5.08 (0.03)[a]	5.11 (0.03)[a]	5.06 (0.11)[a]	4.95 (0.06)[a]
	**Final (mM)**	1.42 (0.03)[a]	1.42 (0.03)[a]	2.40 (0.03)[a]	2.26 (0.21)[a]
***Bacillus* sp. DNRA2 incubation with headspace replenishment (Fig. 5)**	**Initial (mM)**	5.06 (0.09)[a]	-	-	5.04 (0.69)[a]
	**Final (mM)**	0.28 (0.05)[a]	-	-	0.69 (0.29)[a]

[a]The values in the parentheses are the standard deviations of the measurements from biological triplicates

Table S3. Primer sets used for RT-qPCR analyses

Primer set	Sequence	Target gene	Amplicon length (bp)	Slope	*y*-intercept	Amplification efficiency (%)	R^2
Bacillus_nrfAf	5’-GAGTTCAGCGGGATTCCATA-3’	*nrfA*	163	-3.365	38.679	98.2	0.998
Bacillus_nrfAr	5’-TACAGCCTGCTTCGCTTTTT-3’						
Bacillus_recAf	5’-TGAAGTTCAAGCTGCTGGTG-3’	*recA*	155	-3.467	38.652	94.3	0.997
Bacillus_recAr	5’-CAAGTGCTTCAGCGATTTCA-3’						
Bacillus_nosZf	5’-CACGGTGTTGATGTGACTCC-3’	*nosZ*	181	-3.374	34.907	97.888	0.995
Bacillus_nosZr	5’-GATCAACTTCGCGTTCCATT-3’						

Reference

1. Yoon S, Cruz-García C, Sanford R, Ritalahti KM, Löffler FE. 2015. Denitrification versus respiratory ammonification: environmental controls of two competing dissimilatory NO_3^-/NO_2^- reduction pathways in *Shewanella loihica* strain PV-4. *ISME J.* 9:1093-1104.

2. Florindo C, Ferreira R, Borges V, Spellerberg B, Gomes JP, Borrego MJ. 2012. Selection of reference genes for real-time expression studies in *Streptococcus agalactiae. J Microbiol Methods* 90:220-7.

3. Marco ML, Kleerebezem M. 2008. Assessment of real-time RT-PCR for quantification of *Lactobacillus plantarum* gene expression during stationary phase and nutrient starvation. *J Appl Microbiol* 104:587-94.
